# A severe form of Ellis-van Creveld syndrome caused by novel mutations in *EVC2*

**DOI:** 10.1038/s41439-019-0071-9

**Published:** 2019-08-26

**Authors:** Ikuko Ohashi, Yumi Enomoto, Takuya Naruto, Yoshinori Tsurusaki, Yukiko Kuroda, Hiroshi Ishikawa, Makiko Ohyama, Noriko Aida, Gen Nishimura, Kenji Kurosawa

**Affiliations:** 1 Division of Medical Genetics, Kanagawa Children’s Medical Center, Yokohama, Japan; 2 Clinical Research Institute, Kanagawa Children’s Medical Center, Yokohama, Japan; 3 Department of Obstetrics and Gynecology, Kanagawa Children’s Medical Center, Yokohama, Japan; 4 Department of Neonatology, Kanagawa Children’s Medical Center, Yokohama, Japan; 5 Department of Radiology, Kanagawa Children’s Medical Center, Yokohama, Japan; 6 Intractable Disease Center, Saitama Medical University Hospital, Saitama, Japan

**Keywords:** Genetics research, Genetic counselling

## Abstract

Ellis-van Creveld syndrome (EvC MIM. #225500) is an autosomal recessive skeletal dysplasia characterised by thoracic hypoplasia, cardiac anomalies, acromesomelic limb shortening, and postaxial polydactyly. Affected individuals commonly manifest with cardiorespiratory failure as neonates but generally survive neonatal difficulties. We report here on affected Japanese sibs with a lethal phenotype of EvC caused by novel compound heterozygous mutations of *EVC2*, c.871-3 C > G and c.1991dupA.

Ellis-van Creveld syndrome (EvC MIM. #225500) is an autosomal recessive skeletal disorder. The skeletal manifestations of the disorder include thoracic hypoplasia, disproportionate short stature with acromesomelic limb shortening, and postaxial polydactyly. Cardiac anomalies and oral abnormalities are common extraskeletal features^1,2^. Biallelic mutations in *EVC*^3^, *EVC2*^4^, *DYNC2LI1*^5^, *GLI1*^6^, and *WRD35*^7^ have been reported in EvC. However, most affected individuals have *EVC* or *EVC2* mutations. The encoding proteins constitute a protein complex expressed in the basal primary cilia. Biallelic loss of function mutations in *EVC* or *EVC2*^3,4^ lead to primary ciliary dysfunction. In this regard, EvC belongs to a large group of skeletal ciliopathies.

The clinical outcomes of EvC are variable according to the severity of thoracic hypoplasia and concurrent congenital heart defects^8^. Cardiorespiratory failure is common as a neonate. However, most affected individuals survive the neonatal period. Prenatal demise with severe skeletal phenotypes is not unusual^9^. We report here on affected Japanese sibs with a lethal phenotype of EvC caused by novel compound heterozygous mutations of *EVC2*, c.871-3 C > G and c.1991dupA.

Patient 1 was the second child of nonconsanguineous Japanese parents. The first child of the parents was born at 24 weeks of gestation and died soon after birth. The detailed clinical information of the first infant was limited but indicated bilateral polydactyly and hypoplastic thorax. Because of the narrow chest, atrioventricular septum defect, coarctation of aorta, and polyhydramnios in patient 1, the mother was referred to our hospital at 28 weeks of gestation. The proband was born at 39 weeks of gestation with a birth weight of 2899 g (−0.3 SD), length of 46 cm (−1.4 SD), occipitofrontal circumference (OFC) 34 cm ( + 0.5 SD), and chest circumstance 26 cm (−4.3 SD). Apgar scores were 2 and 6 at 1 and 5 min, respectively. He was able to cry at birth but required mechanical ventilation for severe respiratory insufficiency. The patient died 2 h after birth. He had disproportionate short limb dwarfism, postaxial polydactyly, hypoplastic nails, labiogingival frenula, congenital teeth, hypoplastic penis, and hydrocele testis. A skeletal survey showed narrow thorax, normal spine, flared ilia with trident acetabula, acromesomelic shortening of the limbs, bulbous ends of the long bones, and severe brachydactyly (Fig. 1). The clinical and radiological manifestations were suggestive of Ellis-van Creveld syndrome, yet thoracic hypoplasia was unusually severe.

**Fig. 1:**
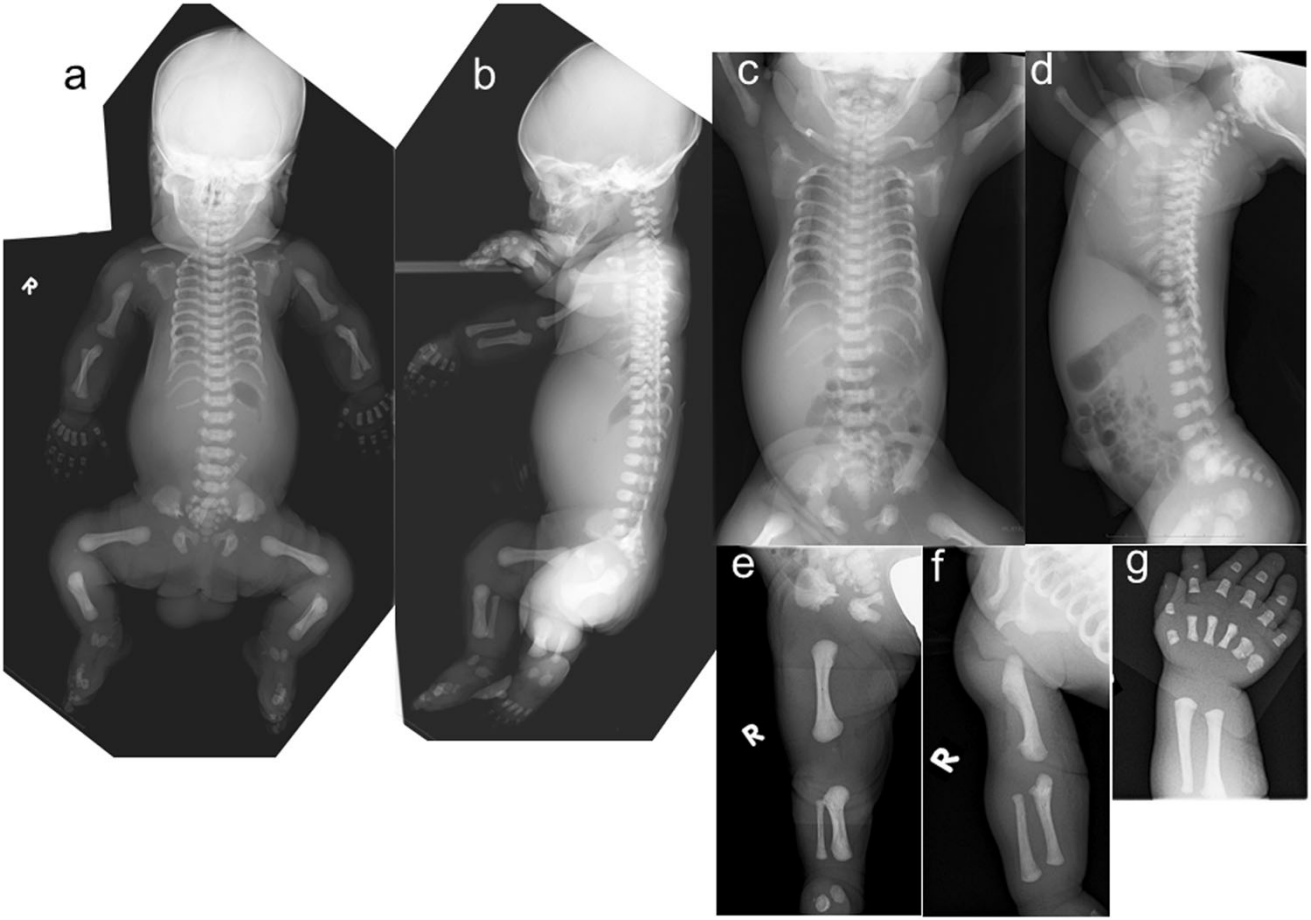
Skeletal images of patient 1 (a, b) and patient 2 (c, d, e, f, g). Both patients showed severe thoracic hypoplasia, acromesomelic shortening of the limbs, flared iliac wings with trident acetabula, and bilateral postiaxial polydactyly. Brachydactyly was very severe. The long bones show bulbous metaphyses. The distinctive shape of the ilia, medial bowing of the humeri and a chicken drumstick appearance of the ulnae and radii are characteristic of EvC

Patient 2 was the third child of 3 siblings born to the parents. He was delivered at 39 weeks of gestation with a birth weight of 2804 g (−0.8 SD), length of 41.5 cm (−4.5 SD), and an OFC of 35.5 cm ( + 1.6 SD). Apgar scores were 7 and 8 at 1 and 5 min, respectively. After birth, he was noted to have a narrow thorax, short limbs and bilateral polydactyly of the hands. Shortly after birth, he developed respiratory distress and was supported with nasal continuous positive air pressure and oxygen supplementation. Postnatal echocardiography validated the partial atrioventricular septal defect. The patient died 2 days after birth due to respiratory failure. A full skeletal survey showed a skeletal phenotype identical to that of patient 1 (Fig. 1). In addition, there were distinctive changes characteristic of EvC, medial bowing of the humeri and drumstick appearance of the ulnae and radii (broad proximal end and narrow distal end of the ulnae and broad distal end, and narrow proximal end of the radii).

Written informed consent was obtained from the parents of the patients in accordance with the Kanagawa Children’s Medical Center Review Board and Ethics Committee. A custom HaloPlex panel was designed using the Agilent SureDesign tool (www.agilent.com/genomics/suredesign) to capture all exons and 10 bp of intronic flanking regions of 29 genes, including *EVC* and *EVC2*. The data analysis, including data alignment, variant calling, and annotation, was performed as described previously^10,11^. Among the filtered variants, novel compound heterozygous variants NM_147127.5:c.871-3 C > G (intron 7) and c.1991dupA (exon 13):[p.(Lys665Glufs*10)] were detected in *EVC2* of both patients 1 and 2. Based on analysis using Mutation Taster (http://www.mutationtaster.org/) and splice site prediction (http://www.fruitfly.org/seq_tools/splice.html), both variants are ‘damaging’. Sanger sequencing confirmed that c.871-3 C > G was derived from the father and c.1991dupA from the mother (Fig. 2).

**Fig. 2:**
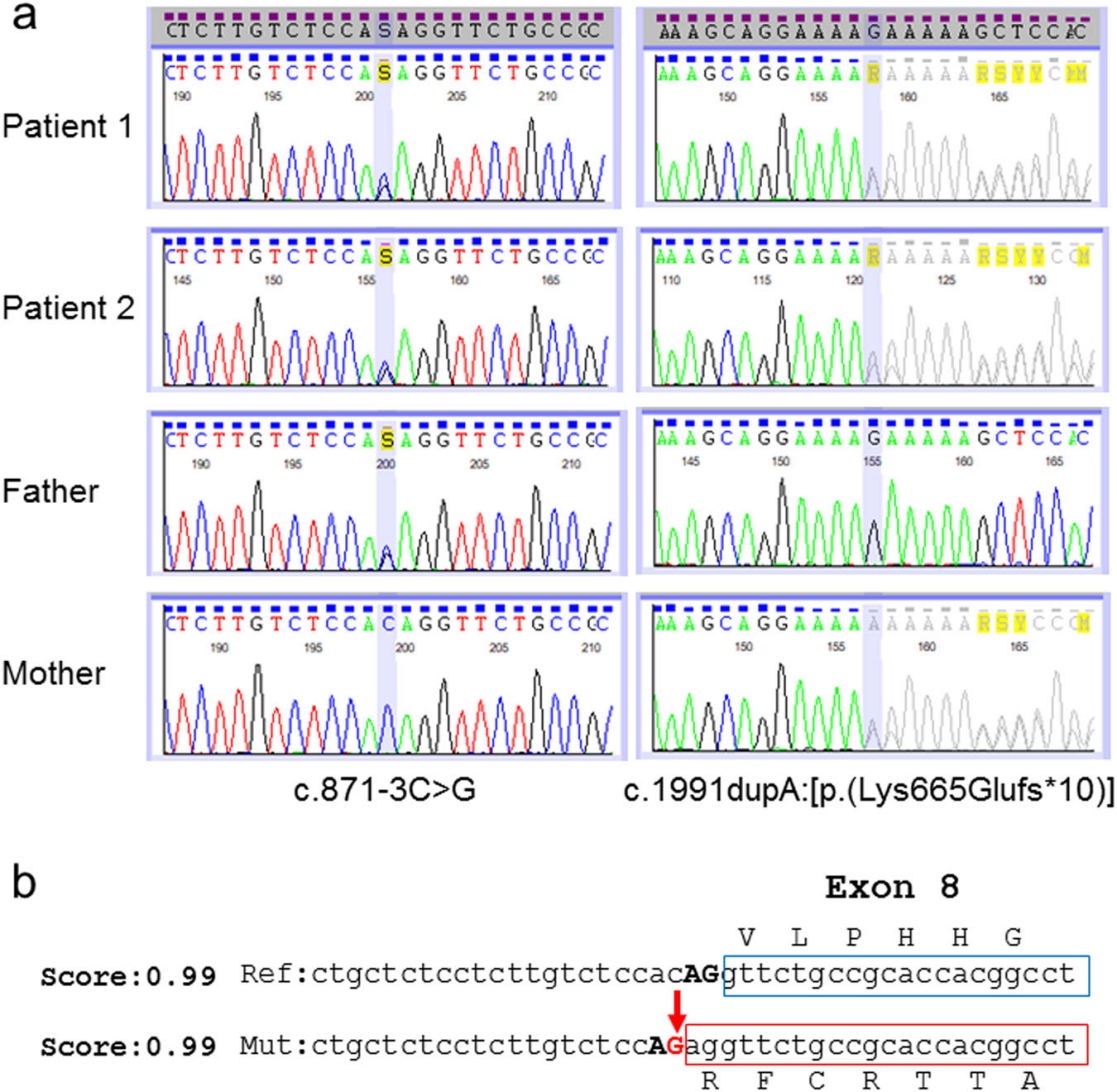
Compound heterozygous variants *EVC2* cause Ellis-van Creveld syndrome in the sibs. **a** Familial electrophoregram shows biallelic mutations, c.871-3 C > G and c.1991dupA. **b**, c.871-3 C > G is highly likely to cause a splicing error because the splice prediction score of the variant is 0.99. The c.871-3 C > G change is predicted to produce a new cryptic splice site, resulting in an aberrant transcript

We identified compound heterozygous variants, c.871-3 C > G and c.1991dupA:[p.(Lys665Glufs*10)] of *EVC2* in siblings with a lethal phenotype of EvC. The former is likely to cause a splicing error on the boundary between intron 7 and exon 8, and the latter to produce an aberrant transcript resulting in premature termination. Both *EVC2* variants are regarded as truncating mutations, as were those previously reported.

The skeletal phenotype of the sibs was very severe. The severe thoracic hypoplasia that caused early demise was reminiscent of severe asphyxiating thoracic dystrophy (ATD) or lethal short rib polydactyly syndromes (SRPS)^9^. However, both patients showed skeletal changes characteristic of EvC, including a distinctive shape of the ilia, medial bowing of the humeri, and drumstick appearance of the ulnae and radii. Genotype-phenotype correlation in EvC remains to be elucidated in EvC. However, there is a report that phenotypic variability is related to variable alternative transcripts of *EVC/EVC2* mutations in different tissues^12^. Unfortunately, we were not able to assess the pattern of transcripts from both abnormal alleles.

In conclusion, we identified novel loss-of-function mutations in the Japanese family, including siblings with a lethal phenotype for EvC. The skeletal changes were consistent with EvC but overlapped with those of severe ATD or SRPS. Further analyses of the transcript patterns of *EVC2* and *EVC* in patients with EvC are needed to facilitate the genotype-phenotype correlation of the disorder.

## Data Availability

The relevant data from this Data Report are hosted at the Human Genome Variation Database at 10.6084/m9.figshare.hgv.2609, 10.6084/m9.figshare.hgv.2612.
